# An Overview Of The Mental Health Problems Among Elite Athletes – Is It A Dream Or A Nightmare?

**DOI:** 10.1192/j.eurpsy.2022.901

**Published:** 2022-09-01

**Authors:** O. Nombora, L. Lopes, L. Santa Marinha

**Affiliations:** 1 Vila Nova de Gaia Hospital Center, Psychiatry And Mental Health Service, Vila Nova de Gaia, Portugal; 2 Vila Nova de Gaia/Espinho Hospital Center, Portugal, Psychiatry And Mental Health Service, Vila Nova de Gaia, Portugal

**Keywords:** Mental Health Problems, sports activities, mental health promotion, elite athletes

## Abstract

**Introduction:**

Mental health problems (MHP) are common among elite athletes (EA) and have received increased attention recently, revealing the need to assess them properly. Although EA are increasingly speaking out on their own MHP in public, research-informed approaches for practitioners are still lacking.

**Objectives:**

We aim to perform an overview of the MPH among EA, emphasizing the potential risk factors and interventions.

**Methods:**

We conduct a non-systematic review of the recent evidence on the topic using PubMed/Medline database.

**Results:**

Although EA have comparable prevalence rates of MHP to the general population, they are exposed to various sports-related stressors. Studies reveal that 50% of EA face MHP during their career, with onset peak around 19 years. Therefore, there is a need for early detection and intervention. Burnout, alcohol abuse, anxiety, depression, insomnia and eating disorders are some MHP reported. Their management should address psychosocial and environmental aspects. Psychoeducation and psychotherapy are considered the first line treatment. Moreover, EA may encounter barriers to seeking mental healthcare. Therefore, it is important to promote positive attitudes about MHP, create an environment that supports mental well-being, resilience, psychological flexibility, self-compassion and coping skills. Screening tools may facilitate the process, so there is a need for validated athlete-specific questionnaires for MHP screening and measuring.

**Conclusions:**

Mental health is an integral dimension of EA wellbeing and performance and should be assessed. Specific programs to support EA mental health are recommended and research targeting common MHP for athletes are needed to better understand how to minimize their distress.

**Disclosure:**

No significant relationships.

